# The Effect of Ostrich Oil on Pain, Healing, and Recurrence of Chronic Anal Fissure: A Randomized Controlled Double‐Blind Clinical Trial

**DOI:** 10.1002/hsr2.72372

**Published:** 2026-06-26

**Authors:** Masoumeh Taghizadeh, Zahra Kourki Nejad Gharaee, Gholamreza Bazmandegan, Zahra Kamiab, Mohammad Ali Zakeri, Xiao Xu, Inshal Jawed, Mohammadreza Gholamrezapour, Ali Mohammad Madahian

**Affiliations:** ^1^ Department of Surgery, School of Medicine, Ali‐Ibn Abi‐Talib Educational and Treatment Hospital Rafsanjan University of Medical Sciences Rafsanjan Iran; ^2^ Non‐Communicable Diseases Research Center Rafsanjan University of Medical Sciences Rafsanjan Iran; ^3^ Physiology‐Pharmacology Research Center, Research Institute of Basic Medical Sciences Rafsanjan University of Medical Sciences Rafsanjan Iran; ^4^ Department of Community Medicine, School of Medicine Rafsanjan University of Medical Sciences Rafsanjan Iran; ^5^ Nursing Research Center Kerman University of Medical Sciences Kerman Iran; ^6^ Clinical Research Development Unit, Ali‐Ibn Abi‐Talib Hospital Rafsanjan University of Medical Sciences Rafsanjan Iran; ^7^ Department of Nursing Nantong Health College of Jiangsu Province Nantong China; ^8^ Dow Medical College Karachi Pakistan; ^9^ Social Determinants of Health Research Centre Rafsanjan University of Medical Sciences Rafsanjan Iran; ^10^ Department of Internal Medicine, School of Medicine, Ali‐Ibn Abi‐Talib Hospital Rafsanjan University of Medical Sciences Rafsanjan Iran

**Keywords:** anal fissure, bleeding, ostrich oil, pain, ulcer

## Abstract

**Background and Aims:**

An anal fissure is a tear in the anal canal below the dentate line, affecting one in five people at some point in their lives. Researchers continuously seek new medications for treating this condition. Ostrich oil has shown anti‐inflammatory and analgesic effects on wounds, as well as angiogenic and re‐epithelialization properties. This study aims to evaluate the effect of ostrich oil on pain reduction and healing in patients with chronic anal fissure (CAF), with implications for improving home‐based palliative care strategies.

**Method:**

In this clinical trial, 150 patients with CAF were randomly divided into intervention and glyceryl trinitrate (GTN) therapy groups. The intervention group received a combination of GTN 0.2% and ostrich oil 50%, while the GTN therapy group received 0.2% GTN ointment until recovery. Patients were evaluated for visual analog scale (VAS), bleeding, healing, and recurrence with a checklist.

**Results:**

Results showed that ostrich oil significantly reduced pain scores compared to GTN over time (group × time interaction: *F* (3,444) = 11.35, *p* < 0.001), with lower pain at all post‐intervention assessments (all *p* < 0.001). Although ulcer grade and bleeding severity were similar immediately post‐treatment, the ostrich oil group demonstrated superior long‐term outcomes at 2‐ and 4‐month follow‐ups, including fewer Grade 3 ulcers, less bleeding, and lower recurrence rates (all *p* < 0.05). Ostrich oil thus provided more effective and sustained relief than GTN for pain management and ulcer healing.

**Conclusion:**

The results of this study suggest that ostrich oil may reduce pain, accelerate healing, shorten the treatment duration, and prevent recurrence of CAF. Given its ease of application and favorable safety profile, ostrich oil could be considered a valuable component of home‐based palliative care strategies for managing CAF.

**Trial Registration:** Iranian Registry of Clinical Trials (IRCT): IRCT20190128042525N4.

**Abbreviations**
CAF: chronic anal fissure
FGF‐2: fibroblast growth factor 2
GTN: glyceryl trinitrate
IBD: inflammatory bowel disease
LIS: lateral internal anal sphincterotomy
NRS: Numeric Pain Rating Scale
STDs: sexually transmitted diseases
TGF‐β1: transforming growth factor beta 1
VAS: visual analog scale
VEGF: vascular endothelial growth factor

## 1 Introduction

Anal fissure is a common disorder experienced by one in five individuals during their lifetime [1]. It is defined as a tear in the anal canal below the dentate line, causing severe pain that is not proportionate to the size of the fissure [2]. Anal fissure typically occurs in young adults and middle‐aged individuals, with an equal prevalence in both genders [3]. A fissure is considered chronic if it persists for more than 8 weeks and is accompanied by edema and fibrosis [2]. Chronic anal fissure (CAF) is characterized by an advanced ulcer observed with white fibers of the internal anal sphincter at the base of the ulcer, fibrosis at the edges of the fissure, skin tags, and hypertrophied papillae in the anal canal [2]. Pathological factors contributing to anal fissure include passage of hard and bulky stools or sudden passage of watery stools, which can lead to fissure formation in the anoderm [4]. Spasm and hypertonia of the internal anal sphincter, along with local ischemia of the anoderm, are two factors responsible for chronic fissure development [5].

The characteristic symptom of anal fissure is pain during defecation, often described as tearing or cutting, accompanied by rectal bleeding [6]. Patients may also complain of intense anal pain and spasm that persists for several hours after bowel movements [1]. Most anal fissures occur near the posterior midline (approximately 85%–90% of cases), with 10%–15% occurring near the anterior midline, and less than 1% occurring outside the midline. Patients are often very sensitive and may not tolerate digital rectal examination, anoscopy, or proctoscopy well [1].

In the treatment of anal fissures, the focus is on breaking the cycle of pain, spasm, and ischemia, which appear to be responsible for the persistence and progression of the fissure [7]. Although acute anal fissures can often be controlled with conservative treatments such as laxatives and local hygiene measures, the treatment of chronic fissures is typically more challenging. Chronic fissures are often managed with topical pharmacological treatments, but recurrence and side effects have led lateral internal anal sphincterotomy (LIS) to become the treatment of choice for chronic fissures [3, 5]. Despite its efficacy, LIS is associated with complications. The most common and concerning complication of LIS is fecal incontinence, which occurs transiently in many patients and permanently in 3% of patients, with more than 6% experiencing varying degrees of fecal incontinence post‐surgery [8]. In the majority of patients, the initial complications include pain and rectal bleeding during and/or after defecation [9]. Other significant complications reported include constipation, itching, perianal discharge, hematoma, fistula, and abscess [8, 10].

The possibility of treatment failure and postsurgical complications has prompted research into nonsurgical treatments for CAF [11], with the primary goal of reducing inflammation, pain, and sometimes spasm of the internal anal sphincter [12]. The use of complementary medicine, such as traditional Iranian medicine, alongside conventional treatments has been recommended. For instance, Avicenna, a prominent figure in Iranian traditional medicine, advocated the topical application of animal oils such as those from chicken, goat, duck, and camel for chronic wounds [13, 14].

For centuries, ostrich oil has been utilized by Egyptians, Africans, and Romans for treating burns and bedsores, significantly improving the healing of thermal burn patients [15]. A laboratory study has demonstrated that ostrich oil possesses radical‐scavenging and anti‐inflammatory properties that promote wound healing and play a significant role in cellular growth, division, and overall cellular integrity [16]. A review article has reported that the topical application of essential fatty acids accelerates wound healing by preserving skin structural integrity, attributed to their rich content of vitamins and amino acids [17]. African ostrich oil comprises approximately 70% fatty acids, including omega‐3 (linolenic acid), omega‐6 (linoleic acid), and omega‐9 (oleic acid) [18]. Linoleic acid, in particular, recognized for its antioxidant and anti‐inflammatory effects, has demonstrated notable efficacy in enhancing wound closure during the early phases of healing in various experimental studies [19]. The moisturizing effect of ostrich oil on epidermal burns has also been well‐documented in animal models [20, 21]. Despite these promising effects on burn treatment and cutaneous wound healing [15], limited research has specifically examined its impact on CAF. A comprehensive literature review indicates that existing evidence regarding ostrich oil is confined to cutaneous wounds in animal models (e.g., mice, dogs) [20, 21, 22] or in vitro studies [15, 16, 18]. To date, no randomized controlled trial (RCT) in humans has been published evaluating the efficacy of ostrich oil for anal fissure. Therefore, this study was designed to investigate the effect of ostrich oil on pain reduction and healing progression in patients with CAF, with the aim of developing evidence‐based therapeutic interventions for pain relief and improved management of this condition.

## 2 Methods

### 2.1 Study Design and Participants

This randomized double‐blind clinical trial was conducted on 150 patients with CAF attending the healthcare clinic in Rafsanjan County over a period of 10 months. The healthcare clinic in Rafsanjan County is a prominent center serving approximately 3000 patients monthly on an outpatient basis in one of the southeastern provinces of Iran. Inclusion criteria for patient enrollment included: age between 18 and 79 years, patients diagnosed with CAF without known causes, presence of symptoms such as pain or bleeding during defecation persisting for at least 8 weeks, and the presence of a posterior or anterior midline ulcer in the anorectal region with associated skin tags.

Patients with other anorectal diseases such as hemorrhoids, fistulas, and abscesses, sexually transmitted diseases (STDs) like AIDS and syphilis, Crohn's disease, anorectal neoplasms, leukemia, history of anal surgery, and previous medical treatment for CAF were excluded from the study. Criteria for patient withdrawal included sensitivity to ostrich oil, irregular use of prescribed medication, and unwillingness to continue participation in the study.

The diagnosis of chronic fissure was made according to the American Society of Colon and Rectal Surgeons (ASCRS). According to the ASCRS clinical practice guidelines, the diagnosis of CAF is primarily clinical and relies on the integration of clinical findings, including: (a) history: severe pain during defecation persisting for more than 6 weeks; (b) physical examination and/or anoscopy: visualization of a longitudinal ulcer typically located in the midline (posterior or anterior); and (c) secondary stigmata of chronicity, when present, such as a sentinel pile (skin tag), hypertrophied anal papilla, or exposed internal sphincter fibers at the base of the fissure [10, 23].

### 2.2 Sample Size

Based on the results of the study by Joda and Al‐Mayoof and the formula below, a sample size of 67 participants was calculated for each group. Taking into account a 20% dropout probability, a total sample size of 152 participants was estimated [24].

$$n=2\frac{{\left(z_{1-\frac{\alpha }{2}}+z_{1-\beta }\right)}^{2}\bar{p}\bar{q}}{{\left(p_{1}-p_{2}\right)}^{2}}.$$

$$\alpha =0.05\to Z_{1-\frac{\alpha }{2}}=1.96.$$

$$\beta =0.20\to Z_{1-\beta }=0.84.$$

**Figure 1:**
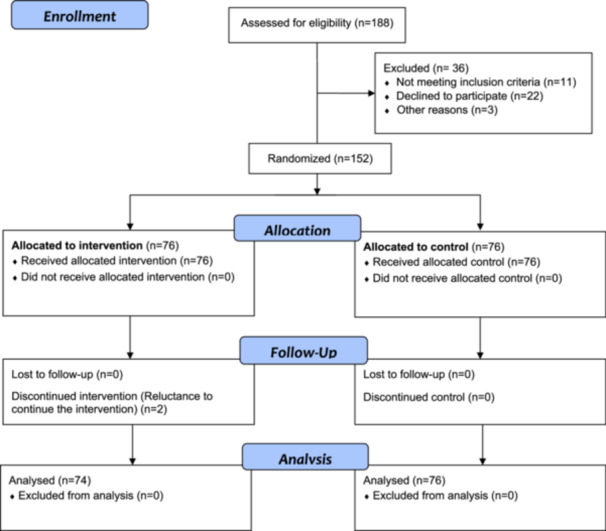
The flow diagram of the study.

P1 = 82% estimated frequency of ulcer healing in the intervention group; P2 = 60% estimated frequency of ulcer healing in the GTN therapy group. Patients were randomly assigned using simple randomization (random number table) to either the intervention or GTN therapy group. Two patients from the intervention group were excluded from the study due to unwillingness to continue the intervention. No adverse events were observed in either group throughout the study period. A total of 74 patients were included in the intervention group, and 76 patients were included in the GTN therapy group for final analysis (Figure 1).

### 2.3 Measurements

#### 2.3.1 Demographic and Clinical Forms

The designed checklist includes demographic and clinical information such as age, gender, duration of illness (months), and primary complaints of the patient, which include sensations of burning, presence of bleeding, and itching.

#### 2.3.2 Numeric Pain Rating Scale (NRS)

NRS is a method for measuring pain intensity in adolescents and adults. One of the methods is the visual analog scale (VAS), which is used to detect relatively small changes in pain levels. In this study, patient pain was assessed using the VAS, where scores ranging from 0 to 10 were assigned. A score of 0 indicated no pain during defecation, while a score of 10 represented severe pain [25]. Patients were asked to report their pain on a scale from 0 to 10.

#### 2.3.3 The Severity of Fissure Anal Bleeding

a) Severity of bleeding: The severity of bleeding in anal fissures is classified into three grades: Grade I (no bleeding during defecation), Grade II (occasional bleeding during defecation), and Grade III (persistent bleeding during defecation).
b) Ulcer grade: Fissures are categorized into Grade I (fresh ulcer with inflammation), Grade II (ulcer with granulation tissue), and Grade III (ulcer covered with complete epithelial tissue). Complete epithelialization of the ulcer observed during physical examination is considered as complete healing of CAF [26].

#### 2.3.4 Time to Remove Symptoms

After achieving complete healing, the duration for resolution of symptoms was recorded, and medical treatment was discontinued (Months 2 and 4). Patients in both intervention and GTN therapy groups who did not show improvement within Month 2 experienced side effects of the drug, such as severe headaches or local complications, or opted for surgical treatment and underwent LIS surgery candidacy.

### 2.4 Data Collection

After obtaining the necessary licenses, the researcher visited the Rafsanjan Health Clinic. Following a medical history and physical examination conducted by a specialist general surgeon, eligible individuals among the clinic's attendees were identified. After explaining the study objectives, obtaining informed consent, and ensuring appropriate compliance based on medical history, patients were enrolled in the study. The demographic and clinical data of patients meeting the inclusion criteria and consenting to participate were reviewed. Opaque sealed envelopes stored with pharmacy. After consent, research nurse opened envelope matching participant ID to dispense preprepared tube. Participants, clinicians, and outcome assessors remained blinded.

Eligible participants were recruited by two nurses employed at the health center. Both groups of patients received similar nutritional recommendations, emphasizing a high‐fiber diet including fruits and vegetables. Initially, demographic and clinical characteristics of patients were recorded. Their fissures, along with the extent of ulceration and bleeding, were graded according to a classification system. Pain severity in patients was assessed using the VAS and recorded. Treatment duration was standardized to 4 months for all participants, regardless of healing status. Follow‐up assessments occurred at immediately after the intervention, Months 2 and 4 post‐intervention by a physician. Disease recurrence was defined as the return of symptoms or the occurrence of diagnostic criteria during clinical examination. Data collection spanned from November 15, 2020 to April 15, 2021.

### 2.5 Intervention

The study intervention was conducted at the Rafsanjan Health Center. Prior to the commencement of the intervention, both intervention and GTN therapy groups received training on how to use and apply the cream. Additionally, the procedure for administering the intervention was explained and demonstrated separately for male and female participants by a male and a female nurse. The intervention group received a 100‐gram container containing glyceryl trinitrate 0.2%–ostrich oil 50% OINT, while the GTN therapy group received an identical‐looking 100‐gram container containing glyceryl trinitrate 0.2% OINT. Participants applied 1 cm ointment ribbon intra‐anally using provided single‐use applicator nozzle (inserted 2–3 cm), twice daily after defecation/sitz bath. In both groups, no systemic analgesics, calcium channel blockers, or botulinum toxin were permitted. Patients and the researching physician were blinded to the administered medication (double‐blind). During the study period, patients were visited by the physician every 14 days, and data related to pain, bleeding, and fissure healing were recorded. All participants received standardized conservative care: psyllium husk 10 g/day, fluid intake ≥ 1.5 L/day, and sitz baths 2×/day. These were identical across groups to minimize confounding.

### 2.6 Data Analysis

For the statistical analysis, the statistical software SPSS version 24.0 for Windows (IBM SPSS Inc., Chicago, IL, USA) was used. Categorical outcomes (e.g., ulcer grade [Grade 2 vs. Grade 3], bleeding severity [Grade 1 vs. Grades 2 + 3], complete healing, and recurrence) were compared between groups using Fisher's exact test (preferred for small expected cell frequencies) or chi‐square test, with odds ratios (OR) and 95% confidence intervals (CI) reported. Effect sizes for proportions were quantified using Cohen's *h* (arcsine‐transformed difference; approximate interpretation: |0.2| small, |0.5| medium, |0.8| large). For the continuous outcome (symptom severity/pain score), between‐group differences at individual time points were assessed using independent samples *t*‐tests. Longitudinal data were analyzed via repeated measures analysis of variance (RM‐ANOVA), with group as the between‐subjects factor and time (baseline, immediate post‐intervention, 2 months, 4 months) as the within‐subjects factor. Mauchly's test was used to assess sphericity; if violated, the Greenhouse–Geisser correction was applied. All hypothesis tests were two‐sided with a prespecified significance level of *α* = 0.05. No adjustment for multiple comparisons was applied to secondary outcomes due to their exploratory/supportive nature.

## 3 Results

A total of 150 patients were enrolled in this RCT trial, with 74 allocated to the ostrich oil group and 76 to the GTN therapy group. Baseline demographic and clinical characteristics are summarized in Table 1. The mean age was 36.77 ± 11.22 years in the ostrich oil group and 35.95 ± 13.51 years in the GTN group (*p* > 0.05, independent *t*‐test). The majority of participants were female (95.9% in ostrich oil vs. 93.4% in GTN; *p* = 0.719, Fisher's exact test). No significant differences were observed between groups in disease duration (*p* > 0.05, chi‐square test), chief complaints (all *p* > 0.05, Fisher's exact test), pre‐intervention ulcer grade (*p* = 0.120, Fisher's exact test; chi‐square; *p* > 0.05), or bleeding severity (*p* > 0.05, chi‐square test). All baseline variables were comparable (*p* > 0.05).

**Table 1:** Baseline demographic and clinical characteristics of participants in the ostrich oil and GTN therapy groups (*N* = 150).

Variable	Ostrich oil (*n* = 74)	GTN therapy (*n* = 76)	*p*
Age (years), mean ± SD	36.77 ± 11.22	35.95 ± 13.51	> 0.05*
Gender, *N* (%)			> 0.05**
Female	71 (95.9)	71 (93.4)	
Male	3 (4.1)	5 (6.6)	
Disease duration (months), *N* (%)			> 0.05**
1–6	44 (59.4)	48 (63.2)	
7–12	15 (20.3)	9 (11.8)	
13–24	6 (8.1)	12 (15.8)	
> 24	9 (12.2)	7 (9.2)	
Chief complaint, *N* (%)			
Pain	71 (95.9)	76 (100.0)	> 0.05**
Burning sensation	50 (67.6)	42 (55.3)	> 0.05**
Bleeding	55 (74.3)	51 (67.1)	> 0.05**
Itching	4 (5.4)	6 (7.9)	> 0.05**
Ulcer grade before intervention, *N* (%)			> 0.05***
Grade 1	3 (4.1)	0 (0.0)	
Grade 2	66 (89.2)	65 (85.5)	
Grade 3	5 (6.8)	11 (14.5)	
Severity of bleeding before intervention, *N* (%)			> 0.05**
Grade 1	20 (27.0)	25 (32.9)	
Grade 2	37 (50.0)	40 (52.6)	
Grade 3	17 (23.0)	11 (14.5)	

*Note:* Data are presented as mean ± standard deviation (SD) or number (percentage).

* *p* value derived from independent samples *t*‐test.

** *p* value derived from chi‐square test.

*** *p* value derived from Fisher's exact test.

**Figure 2:**
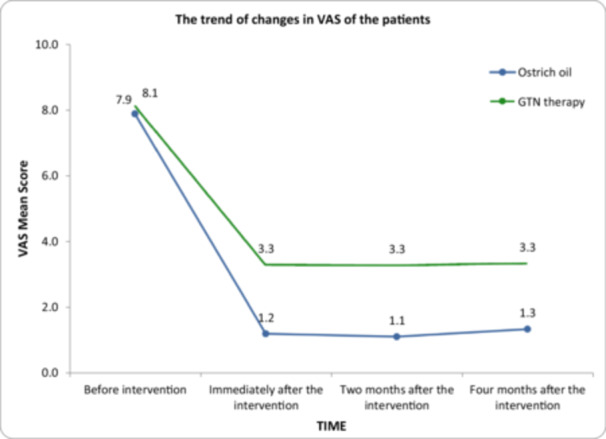
The trend of changes in VAS of the patients over time and between two groups.

Longitudinal changes in symptom severity/pain score (assessed on a continuous scale, e.g., VAS) are presented in Table 2. Repeated measures ANOVA revealed significant main effects for group (*F* (1,148) = 623.55, *p* < 0.001), time (*F* (3,444) = 438.59, *p* < 0.001), and group × time interaction (*F* (3,444) = 11.35, *p* < 0.001). The significant group effect indicated that the overall mean pain score across all time points was lower in the ostrich oil group compared to the GTN group. The time effect reflected a substantial reduction in pain scores over the study period in both groups. The significant interaction demonstrated that the rate of pain reduction differed between groups, with a steeper decline in the ostrich oil group, particularly during the active treatment phase (immediately post‐intervention and at early follow‐up). Post hoc between‐group comparisons confirmed significantly lower pain scores in the ostrich oil group at all post‐intervention time points (all *p* < 0.001, independent *t*‐tests) (Figure 2).

**Table 2:** Mean [Pain/Symptom Severity Score] at baseline and follow‐up in ostrich oil and GTN groups with repeated measures ANOVA.

Variable	Group				Standard error	*p*
	Ostrich oil (*n* = 74)		GTN therapy (*n* = 76)			
	*M*	SD	*M*	SD		
Before intervention	7.88	2.44	8.12	1.74	0.34	> 0.05
Immediately after the intervention	1.18	1.93	3.28	2.63	0.37	< 0.001
2 months after the intervention	1.09	1.96	3.26	2.68	0.38	< 0.001
4 months after the intervention	1.32	2.26	3.32	2.71	0.40	< 0.001
*Repeated measures ANOVA results*						

Source of change	Degrees of freedom		*F*		*p*	
Group	1		623.55		< 0.001	
Time	3		438.59		< 0.001	
Group × time interaction	3		11.35		< 0.001	

*Note:* Values are mean ± standard deviation (SD). Standard error refers to the standard error of the mean difference between groups at each time point. Between‐group *p* values derived from independent samples *t*‐tests. Repeated measures ANOVA (assuming sphericity; if correction applied, e.g., Greenhouse–Geisser, specify accordingly). All post‐intervention comparisons and ANOVA effects were highly significant, consistently favoring the ostrich oil group (lower scores).

Categorical clinical outcomes, including ulcer grade, bleeding severity, complete healing, and recurrence, are detailed in Table 3. Immediately after the intervention, no significant differences were observed in ulcer grade (*p* > 0.05) or bleeding severity (*p* > 0.05) between groups, suggesting comparable initial responses. However, at the 2‐month and 4‐month follow‐ups, the ostrich oil group showed significantly better outcomes: lower proportion of Grade 3 ulcers (*p* < 0.05 at 2 and 4 months), reduced bleeding severity (*p* < 0.05 at 2 and 4 months), and lower recurrence rates (*p* < 0.05 at 2 and 4 months; all Fisher's exact test). Effect sizes (Cohen's *h*) were medium in magnitude for most significant comparisons (ranging from |0.39| to |0.58|), and OR consistently favored the ostrich oil group (detailed in Table 3). At the end of the intervention, one patient (1.4%) in the ostrich oil group and three patients (3.9%) in the GTN group required LIS due to persistent symptoms (*p* > 0.05).

**Table 3:** Categorical clinical outcomes in ostrich oil versus glyceryl trinitrate (GTN) therapy groups at different time points.

Outcome variable	Time point	Ostrich oil (*n* = 74) *N* (%)	GTN (*n* = 76) *N* (%)	Cohen's *h*	Odds ratio (ostrich oil vs. GTN)	95% CI for OR	*p* (Fisher's exact test)
Ulcer grade—Grade 2 (better)	Immediately after	1 (1.4)	3 (3.9)	—	0.34	[0.03–3.32]	> 0.05
	2 months after	5 (6.8)	18 (23.7)	−0.49	0.23	[0.08–0.67]	< 0.05
	4 months after	7 (9.5)	21 (27.6)	−0.48	0.27	[0.11–0.69]	< 0.05
Bleeding severity—Grade 1 (better)	Immediately after	71 (95.9)	71 (93.4)	—	1.67	[0.38–7.28]	> 0.05
	2 months after	71 (95.9)	59 (77.6)	0.58	6.82	[1.91–24.4]	< 0.05
	4 months after	67 (90.5)	58 (76.3)	0.39	2.97	[1.16–7.61]	< 0.05
Complete healing (vs. candidate for LIS)	Immediately after	73 (98.6)	73 (96.1)	—	3.00	[0.30–29.6]	> 0.05
Recurrence	2 months after	11 (14.9)	29 (38.2)	−0.54	0.28	[0.13–0.62]	< 0.05
	4 months after	21 (28.4)	40 (52.6)	−0.50	0.36	[0.18–0.70]	< 0.05

*Note:* Values are presented as number of participants (*N*) and percentage (%). *p* values were derived from Fisher's exact test.

In summary, while immediate post‐intervention effects on categorical healing parameters were similar, the ostrich oil group exhibited superior long‐term outcomes in pain reduction, ulcer healing, bleeding control, and recurrence prevention compared to GTN therapy.

## 4 Discussion

This study aimed to investigate the effect of ostrich oil on pain and the treatment process of CAF. As reviewed in the literature, anal fissure is a common condition treated with glyceryl trinitrate, which, however, may lead to disease recurrence and side effects [3, 5]. Ostrich oil is rich in essential fatty acids, vitamins, and amino acids [17, 27], exerting angiogenic effects [20] and accelerating re‐epithelialization [16].

The results of this study demonstrated that ostrich oil can reduce pain, expedite healing, decrease the severity of ulcer grading and bleeding. Consistent with the findings of the current study, Ahangar and colleagues demonstrated the anti‐inflammatory and analgesic effects of ostrich oil in a study on a new physical hydrogel compound composed of polyvinyl pyrrolidone and chitosan containing ostrich oil on Syrian mice [28]. Additionally, Yagatani and colleagues previously demonstrated in 2003 the role of ostrich oil in inhibiting TNF‐α, an inflammatory cytokine [29]. Reviewing the literature reveals few studies examining the positive effects of ostrich oil on wound healing. Farahpour and colleagues investigated the effect of topical application of ostrich oil on improving *Staphylococcus aureus* and *Pseudomonas aeruginosa*‐infected wounds in a mouse model, observing significant reductions in wound size, angiogenesis, fibroblast migration, and collagen deposition [20]. The immunomodulatory and angiogenic effects of ostrich oil are mediated through the expression of vascular endothelial growth factor (VEGF), transforming growth factor beta 1 (TGF‐β1), and fibroblast growth factor 2 (FGF‐2), which play crucial roles in the wound healing process.

In reviewing and comparing treatments using natural substances for anal fissure, Derakhshan's review explored the impact of topical cream containing clove oil and aloe vera [14]. Elwakeel and colleagues demonstrated the effect of 1% clove oil cream compared to 5% lignocaine cream over a 6‐week period [30]. Renzi and colleagues showed positive effects on anal fissure improvement with 3% Myoxinol ointment (hydrolyzed extract of Hibiscus edible parts) [31]. There is a wide range of management options available for the improvement and treatment of anal fissure, including topical nitrates, calcium channel blockers, botulinum toxin injections, and sphincterotomy [3]. However, each of these methods has associated side effects and challenges. Therefore, attention to natural options such as ostrich oil may have positive effects on anal fissure improvement.

Furthermore, other studies have focused on improving symptoms such as pain and bleeding in patients with anal fissure, which align with the findings of the current study. Rahmani and colleagues demonstrated that topical application of 0.5% aloe vera cream led to improvement in pain, ulcer healing, and bleeding during defecation in the intervention group [26]. Additionally, Salari and colleagues compared the effects of egg yolk and nitroglycerin on acute anal fissure for 10 days and found that egg yolk significantly reduced pain and bleeding in patients [32]. However, attention to treatment duration and follow‐up period are factors that can significantly impact the effectiveness of interventions. In the present study, the effect of ostrich oil should be interpreted as an adjunctive factor, as both groups received glyceryl trinitrate. A review of the existing literature revealed a lack of human studies addressing this specific intervention, highlighting the need for further research. Consequently, the findings should be interpreted with caution.

In the present study, no side effects related to the use of ostrich oil, which acts as a drug carrier enhancing absorption, were observed, including no occurrences of nitroglycerin‐related adverse effects such as headaches. However, in the study by Elwakeel and colleagues, the 1% clove oil cream caused severe itching and burning sensation due to sensitivity to clove oil when compared to the 5% lignocaine cream [30]. Additionally, the topical application of Myoxinol ointment (hydrolyzed extract of Hibiscus) in Renzi and colleagues' study was associated with perianal itching [31]. Attention to the possibility of sensitivities and some reported adverse effects in studies, such as vascular issues [33] and cardiac arrhythmias [34], underscores the need for careful consideration when using natural substances.

### 4.1 Limitations

In this study, a single physician was utilized to assess outcomes in all patients to mitigate variability arising from different physician opinions. However, due to patients' preference for consulting physicians of the same gender, a majority of study participants were female, which prevented us from evaluating the drug's effects between genders. Moreover, the study employed 0.2% nitroglycerin in the GTN therapy group. Due to the limited number of patients eligible for LIS surgery, we could not directly investigate whether ostrich oil reduces the need for LIS surgery in the current disease. Therefore, further studies are necessary in this regard. Additionally, our study used ostrich oil at a concentration of 50%. Determining the optimal dosage of ostrich oil in combination with GTN to maximize its effectiveness in improving anal fissures and potentially preventing disease requires further investigation. We followed up with patients 4 months after complete recovery regarding disease recurrence, suggesting that future studies should be conducted with larger sample sizes and longer follow‐up periods. Molecular studies are also recommended for a more precise assessment of the effect of ostrich oil on anal fissure improvement. This trial evaluates ostrich oil as an adjunct to standard GTN therapy, not as monotherapy. Conclusions about ostrich oil efficacy independent of GTN cannot be drawn. In the present study, the gender imbalance (*N* = 142 female participants) should be acknowledged as a limitation that may compromise the generalizability of findings to male populations. Further research is warranted to examine potential gender‐specific effects of the intervention.

## 5 Conclusion

Our findings indicate that topical adjunctive ostrich oil combined with GTN was associated with higher healing rates and reduced bleeding severity compared to GTN monotherapy. Nevertheless, given that the majority of participants were female and that ostrich oil was administered exclusively as an adjunctive agent rather than as monotherapy, these results warrant validation through additional studies in more representative populations. Furthermore, the therapeutic regimen requires a prolonged treatment duration, which may pose tolerability challenges for certain individuals and potentially compromise treatment adherence and patient satisfaction. Consequently, future research should incorporate extended follow‐up periods in both male and female participants, utilizing larger and more demographically diverse cohorts to enhance the external validity and clinical applicability of the findings.

## Author Contributions

Conceptualization: Masoumeh Taghizadeh, Gholamreza Bazmandegan, Zahra Kamiab, Mohammadreza Gholamrezapour, and Ali Mohammad Madahian. Data curation: Zahra Kourki Nejad Gharaee, Masoumeh Taghizadeh, and Mohammadreza Gholamrezapour. Formal analysis: Zahra Kamiab, Mohammad Ali Zakeri, and Xiao Xu. Methodology: Masoumeh Taghizadeh, Gholamreza Bazmandegan, and Ali Mohammad Madahian. Project administration: Masoumeh Taghizadeh and Ali Mohammad Madahian. Visualization: Masoumeh Taghizadeh, Mohammad Ali Zakeri, Zahra Kamiab, Inshal Jawed, and Xiao Xu. Writing – original draft: Mohammad Ali Zakeri, Zahra Kourki Nejad Gharaee, Inshal Jawed, and Xiao Xu. Writing – review and editing: Mohammad Ali Zakeri, Ali Mohammad Madahian, Inshal Jawed, and Xiao Xu. All authors have read and approved the final version of the manuscript.

## Funding

The authors have nothing to report.

## Disclosure

Ali Mohammad Madahian affirms that this manuscript is an honest, accurate, and transparent account of the study being reported; that no important aspects of the study have been omitted; and that any discrepancies from the study as planned (registered at IRCT.ir: IRCT20190128042525N4 ||http://www.irct.ir/) have been explained.

## Ethics Statement

This study was approved by the Ethics Committee of Rafsanjan University of Medical Sciences (IR.RUMS.REC.1399.162). Prior to commencing the study, the research protocol was approved by the Iranian Registry of Clinical Trials (IRCT) (IRCT20190128042525N4 ||http://www.irct.ir/) with the Clinical Trial Registration Date: November 2, 2020 (https://irct.behdasht.gov.ir/trial/45759). All experiments were performed in accordance with relevant guidelines, regulations, and the Declaration of Helsinki.

## Consent

Before initiating the study, an informed consent form was designed, and patients who met the inclusion criteria were asked to sign this consent form. Before obtaining consent from patients regarding the research objectives, voluntary participation, and confidentiality of information, detailed explanations were provided.

## Conflicts of Interest

The authors declare no conflicts of interest.

## Supporting information

Supporting File

## Data Availability

The data used to support the findings of this study are available from the corresponding author upon request. Ali Mohammad Madahian had full access to all of the data in this study and takes complete responsibility for the integrity of the data and the accuracy of the data analysis.

## References

[1] Y. Lu , M. R. Kwaan , and A. Y. Lin , “Diagnosis and Treatment of Anal Fissures in 2021,” Journal of the American Medical Association 325, no. 7 (2021): 688–689. 33591336 10.1001/jama.2020.16705

[2] A. Gilani and G. Tierney , “Chronic Anal Fissure in Adults,” BMJ 376 (2022): e066834. 35022226 10.1136/bmj-2021-066834

[3] P. A. Boland , M. E. Kelly , N. E. Donlon , et al., “Management Options for Chronic Anal Fissure: A Systematic Review of Randomised Controlled Trials,” International Journal of Colorectal Disease 35 (2020): 1807–1815. 32712929 10.1007/s00384-020-03699-4

[4] S. Chen and Q. Yu , “A New Theory on the Cause of Anal Fissure–Impaction Theory,” Journal of Coloproctology 40, no. 4 (2020): 321–325.

[5] M. Alawady , S. H. Emile , M. Abdelnaby , H. Elbanna , and M. Farid , “Posterolateral Versus Lateral Internal Anal Sphincterotomy in the Treatment of Chronic Anal Fissure: A Randomized Controlled Trial,” International Journal of Colorectal Disease 33 (2018): 1461–1467. 29779044 10.1007/s00384-018-3087-6

[6] V. Wienert , F. Raulf , and H. Mlitz , Anal Fissure: Symptoms, Diagnosis and Therapies (Springer, 2017).

[7] Y. A. Shelygin , O. V. Tkalich , A. A. Ponomarenko , et al., “Follow‐Up Results of Combination Treatment of Chronic Anal Fissure,” International Journal of Pharmaceutical Research (2020): 244.

[8] T. Acar , N. Acar , F. Güngör , et al., “Treatment of Chronic Anal Fissure: Is Open Lateral Internal Sphincterotomy (LIS) a Safe and Adequate Option?,” Asian Journal of Surgery 42, no. 5 (2019): 628–633. 30366766 10.1016/j.asjsur.2018.10.001

[9] R. L. Nelson , “Anal Fissure (Chronic),” BMJ Clinical Evidence 2014 (2014): 0407. PMC4229958 25391392

[10] D. B Stewart Sr., W. Gaertner , S. Glasgow , J. Migaly , D. Feingold , and S. R. Steele , “Clinical Practice Guideline for the Management of Anal Fissures,” Diseases of the Colon and Rectum 60, no. 1 (2017): 7–14. 27926552 10.1097/DCR.0000000000000735

[11] L. F. Tauro , V. V. Shindhe , P. S. Aithala , J. J. S. Martis , and H. D. Shenoy , “Comparative Study of Glyceryl Trinitrate Ointment Versus Surgical Management of Chronic Anal Fissure,” Indian Journal of Surgery 73 (2011): 268–277. 22851840 10.1007/s12262-011-0239-0 PMC3144351

[12] R. L. Nelson , D. Manuel , C. Gumienny , et al., “A Systematic Review and Meta‐Analysis of the Treatment of Anal Fissure,” Techniques in Coloproctology 21 (2017): 605–625. 28795245 10.1007/s10151-017-1664-2

[13] G. Mosleh , M. Nimrouzi , P. Badr , Z. Abolhasanzadeh , A. Azadi , and A. Mohagheghzadeh , “The Approach of Traditional Persian Medicine to Treatment of Anal Fissure,” Traditional and Integrative Medicine 4, no. 2 (2019): 78–83.

[14] A. R. Derakhshan , “Natural Treatments for Fissure in Ano Used by Traditional Persian Scholars, Razi (Rhazes) and Ibn Sina (Avicenna),” Journal of Evidence‐Based Complementary & Alternative Medicine 22, no. 2 (2017): 324–333. 27279645 10.1177/2156587216650302 PMC5871188

[15] U. D. Palanisamy , M. Sivanathan , A. K. Radhakrishnan , N. Haleagrahara , T. Subramaniam , and G. S. Chiew , “An Effective Ostrich Oil Bleaching Technique Using Peroxide Value as an Indicator,” Molecules 16, no. 7 (2011): 5709–5719. 21730920 10.3390/molecules16075709 PMC6264162

[16] D. C. Bennett , W. E. Code , D. V. Godin , and K. M. Cheng , “Comparison of the Antioxidant Properties of Emu Oil With Other Avian Oils,” Australian Journal of Experimental Agriculture 48, no. 10 (2008): 1345–1350.

[17] U. Akbar , M. Yang , D. Kurian , and C. Mohan , “Omega‐3 Fatty Acids in Rheumatic Diseases: A Critical Review,” JCR: Journal of Clinical Rheumatology 23, no. 6 (2017): 330–339. 28816722 10.1097/RHU.0000000000000563

[18] J. Ponphaiboon , S. Limmatvapirat , A. Chaidedgumjorn , and C. Limmatvapirat , “Physicochemical Property, Fatty Acid Composition, and Antioxidant Activity of Ostrich Oils Using Different Rendering Methods,” LWT 93 (2018): 45–50.

[19] N.‐Y. Park , G. Valacchi , and Y. Lim , “Effect of Dietary Conjugated Linoleic Acid Supplementation on Early Inflammatory Responses During Cutaneous Wound Healing,” Mediators of Inflammation 2010 (2010): 1–8. 10.1155/2010/342328 PMC2943105 20871865

[20] M. R. Farahpour , M. Vahid , and A. Oryan , “Effectiveness of Topical Application of Ostrich Oil on the Healing of *Staphylococcus aureus*‐ and *Pseudomonas aeruginosa*‐Infected Wounds,” Connective Tissue Research 59, no. 3 (2018): 212–222. 28682114 10.1080/03008207.2017.1350174

[21] A. Nada , H. AbuAhmed , A. Khafaga , and M. ElKammar , “Clinical and Histopathological Evaluation of the Effectiveness of Lavender Oil Compared With Black Seed Oil, Ostrich Oil and Cod Liver Oil on the Second Intention Wound Healing in Dogs,” Alexandria Journal of Veterinary Sciences 46, no. 1 (2015): 57.

[22] S. M. Alshahrani , “Preparation, Characterization and *In Vivo* Anti‐Inflammatory Studies of Ostrich Oil Based Nanoemulsion,” Journal of Oleo Science 68, no. 3 (2019): 203–208. 30760670 10.5650/jos.ess18213

[23] J. S. Davids , A. T. Hawkins , A. R. Bhama , et al., “The American Society of Colon and Rectal Surgeons Clinical Practice Guidelines for the Management of Anal Fissures,” Diseases of the Colon and Rectum 66, no. 2 (2023): 190–199. 36321851 10.1097/DCR.0000000000002664

[24] A. E. Joda and A. F. Al‐Mayoof , “Efficacy of Nitroglycerine Ointment in the Treatment of Pediatric Anal Fissure,” Journal of Pediatric Surgery 52, no. 11 (2017): 1782–1786. 28410787 10.1016/j.jpedsurg.2017.04.003

[25] A. Williamson , and H. Barbara , “Pain: Areview of Three Commonly Used Pain Rating Scales,” Journal ofclinical nursing 14.7 (2005): 798–804. 10.1111/j.1365-2702.2005.01121.x 16000093

[26] N. Rahmani , M. Khademloo , K. Vosoughi , and S. Assadpour , “Effects of Aloe Vera Cream on Chronic Anal Fissure Pain, Wound Healing and Hemorrhaging Upon Defection: A Prospective Double Blind Clinical Trial,” European Review for Medical and Pharmacological Sciences 18, no. 7 (2014): 1078–1084. 24763890

[27] L. C. Hoffman , M. Joubert , T. S. Brand , and M. Manley , “The Effect of Dietary Fish Oil Rich in n−3 Fatty Acids on the Organoleptic, Fatty Acid and Physicochemical Characteristics of Ostrich Meat,” Meat Science 70, no. 1 (2005): 45–53. 22063279 10.1016/j.meatsci.2004.11.019

[28] N. Ahangar , M. G. Razdari , and P. Ebrahimnejad , “Preparation, Characterization and Analgesic Evaluation of a Novel Physical Hydrogel Composed of Opened‐Ring Poly (Vinyl Pyrrolidone) and Chitosan Containing Ostrich Oil in Mice,” Journal of Mazandaran University of Medical Sciences 28, no. 165 (2018): 1–12.

[29] S. Yoganathan , R. Nicolosi , T. Wilson , et al., “Antagonism of Croton Oil Inflammation by Topical Emu Oil in CD‐1 Mice,” Lipids 38, no. 6 (2003): 603–607. 12934669 10.1007/s11745-003-1104-y

[30] H. A. Elwakeel , H. A. Moneim , M. Farid , and A. A. Gohar , “Clove Oil Cream: A New Effective Treatment for Chronic Anal Fissure,” Colorectal Disease 9, no. 6 (2007): 549–552. 17573751 10.1111/j.1463-1318.2006.01185.x

[31] A. Renzi , A. Brillantino , G. Di Sarno , et al., “Myoxinol (Hydrolyzed *Hibiscus esculentus* Extract) in the Cure of Chronic Anal Fissure: Early Clinical and Functional Outcomes,” Gastroenterology Research and Practice 2015 (2015): 1–4. 10.1155/2015/567920 PMC4378599 25861259

[32] M. Salari , R. Salari , M. Dadgarmoghadam , M. Khadem‐Rezaiyan , and M. Hosseini , “Efficacy of Egg Yolk and Nitroglycerin Ointment as Treatments for Acute Anal Fissures: A Randomized Clinical Trial Study,” Electronic Physician 8, no. 10 (2016): 3035–3041. 27957300 10.19082/3035 PMC5133025

[33] M. A. Zakeri , M. H. Bagheripour , M. Iriti , and M. Dehghan , “Portal Vein Thrombosis After the Consumption of Date Seed Powder: A Case Study,” Case Reports in Medicine 2021 (2021): 1–5. 10.1155/2021/6668722 PMC8075688 33959162

[34] M. A. Zakeri , V. Mohammadi , G. Bazmandegan , and M. Zakeri , “Description of Ventricular Arrhythmia After Taking Herbal Medicines in Middle‐Aged Couples,” Case Reports in Cardiology 2020 (2020): 1–4. 10.1155/2020/6061958 PMC7547334 33062339

